# Dip-Coating Process Engineering and Performance Optimization for Three-State Electrochromic Devices

**DOI:** 10.1186/s11671-017-2163-0

**Published:** 2017-06-06

**Authors:** Lu Wu, Dejiang Yang, Lixun Fei, Yue Huang, Fang Wu, Yiling Sun, Jiayuan Shi, Yong Xiang

**Affiliations:** School of Energy Science and Engineering, University of Electronic Science and Technology of China, 2006 Xiyuan Ave, West High-Tech Zone, Chengdu, 611731 Sichuan People’s Republic of China

**Keywords:** Electrochromism, Electrodeposition, Titanium dioxide nanoparticle, Dip-coating process engineering, Optical performance

## Abstract

Titanium dioxide (TiO_2_) nanoparticles were modified onto fluorine-doped tin oxide (FTO) via dip-coating technique with different nanoparticle sizes, lifting speeds, precursor concentrations, and dipping numbers. Electrodeposition-based electrochromic device with reversible three-state optical transformation (transparent, mirror, and black) was fabricated subsequently by sandwiching a suitable amount of gel electrolyte between modified FTO electrode and flat FTO electrode. Correlation between dip-coating process engineering, morphological features of TiO_2_ thin films, i.e., thickness and roughness, as well as performance of electrochromic devices, i.e., optical contrast, switching time, and cycling stability, were investigated. The modified device exhibits high optical contrast of 57%, the short coloration/bleaching switching time of 6 and 20 s, and excellent cycling stability after 1500 cycles of only 27% decrement rate by adjusting dip-coating processes engineering. The results in this study will provide valuable guidance for rational design of the electrochromic device with satisfactory performance.

**Electronic supplementary material:**

The online version of this article (doi:10.1186/s11671-017-2163-0) contains supplementary material, which is available to authorized users.

## Background

An attractive feature of electrochromic materials is the ability to change their optical properties in a reversible and persistent manner when applied with an electrical voltage. Since the pioneering work of Deb [1], a variety of electrochromic materials have been developed, which can be grouped into several subsets: transition metal oxides [2], Prussian blue [3], conducting polymers [4], viologens [5], transition metal ions coordination compound [6], hybrid electrochromic materials [7], and reversible electrodeposition-based electrochromic materials [8, 9]. Their electrochromic performance including optical contrast, switching time, coloration efficiency, cycling stability, and optical memory effect have been investigated extensively, which promote us to expand the applications of electrochromic materials in the area of smart windows, anti-glare rear-view mirrors, electrochromic display, electronic papers, and military camouflage [10–19]. Electrochromic devices based on reversible electrodeposition are promising for application in light modulations owing to their simple sandwich-type structure and facile and low-cost fabrication. Their optical properties can be manipulated via deposition of metal (copper (Cu), bismuth (Bi), plumbum (Pb), nickel (Ni), silver (Ag), etc.) onto transparent conducting electrodes under an applied electrical voltage and dissolution of metal back into electrolyte upon removal of the voltage [20–26]. Bismuth–copper (Bi/Cu) electrodeposition devices are widely used in information displays due to their rapid and reversible switching between black and transparent states enabled by the oxidation–reduction between Bi and Bi^3+^ [9, 23–25]. Similarly, Ag-based electrodeposition system [26–29] has also been developed for fabricating electrochromic devices for its ability to realize mirror state.

Usually, appropriate electrode surface modification may trigger reversible and multiple color states of the electrodeposition-based electrochromic device due to the absorption and/or multiple scattering of light of the modified electrode surface [30–33]. Various techniques, including sputtering [34], vacuum evaporation [35], chemical vapor deposition [36], hydrothermal [37], electrodeposition [38], and sol–gel [39, 40], have been utilized to fabricate electrochromic thin films. Among various techniques, the sol–gel approach is advantageous due to its low cost, amenable for large area preparation, and easy to handle properties, out of which the spin-coating and dip-coating techniques are widely used. Compared with spin-coating, the dip-coating technique is preferred due to its higher controllability and more applicable to large scale preparation [24]. Moreover, Deepa et al. [24] also reported that dip-coated electrochromic devices based on tungsten trioxide (WO_3_) thin films showed superior performance compared with spin-coated devices, such as improved transmission modulation, coloration efficiency, switching speed, and coloration/bleaching cycles. The dip-coating technique, however, has not yet been applied in the fabrication of electrodeposition-based Ag/Cu electrochromic devices.

Basically, the electrochromic performance (i.e., optical contrast, switching time, coloration efficiency, cycling stability, and optical memory effect) of electrochromic materials basically depends on their structural, surface morphological, and compositional properties [41]. It is thus extremely necessary to have a closer inspection of preparation parameters for the property improvement of electrochromic materials. Deepa et al. [42] fabricated WO_3_ films via dip-coating technique, and the influence of relative humidity (RH) change (55 and 75% RH) during thin film deposition from an oxalato-acetylated peroxotungstic acid sol on the microstructure and electrochromic properties of WO_3_ films obtained upon annealing was presented. Faster switching kinetics between the clear and blue states, a greater current density for lithium intercalation, a higher diffusion coefficient for lithium, and a superior cycling stability, are obtained by the film fabricated under a 75% RH, indicating the effect of humidity change on the structure and electrochromic properties of electrochromic materials. Sun and his co-workers [43] prepared WO_3_ thin films by sol–gel route combined with the spin-coating method. The influence of annealing temperature on microstructure and optical properties of WO_3_ films were investigated, and higher transmittance modulation in the visible range at lower annealing temperature was obtained. The effects of the type and content of organic moiety in the precursor sol, film preparation method (spin- or dip-coating) on film properties have also been investigated extensively [43, 44], to have a general understanding of the correlation between the electrochromic performance and the fabrication parameters of electrochromic thin films. Araki et al. [41] deposited Ag onto a modified indium tin oxide (ITO) electrode via spin-coating and obtained a reversible black and mirror states. Further pursuit of multiple color states has also been carried out by Tsuboi and his co-workers [42, 44] by controlling the growth of Ag grains under different voltages, indicating that the manipulation of the size and shape of nanoparticles can result in dramatic changes in color. In our previous study [33], we fabricated electrodeposition-based Ag/Cu electrochromic device with a reversible three-state optical transformation (transparent, black, and mirror states), with a conducting TiO_2_ nanoparticle-modified fluorine-doped tin oxide (FTO) electrode fabricated via spin-coating technique. We also demonstrated that the optical properties of the device in different states can be controlled effectively by manipulating the surface structure of the TiO_2_-modified FTO electrode. However, the closer inspection of effects of electrode surface modification on the multi-state electrochromic device is rarely reported. Therefore, a thorough investigation on electrodeposition-based electrochromic devices properties through the fabrication parameters is significant.

In this study, TiO_2_ nanoparticles were modified onto FTO via dip-coating technique, followed by sandwiching a suitable amount of gel electrolyte between a modified FTO electrode and a flat FTO electrode to fabricate an electrodeposition-based electrochromic device with reversible three-state optical transformation. For the high controllability of dip-coating technique, the optical performance of devices can be adjusted by manipulating the electrode surface modification. The nanoparticle size is an important parameter that can be manipulated and could make the performance of fabricated devices different. Therefore, the nanoparticle size is adjusted to investigate its effects on the microstructures of TiO_2_ thin films and performance of fabricated devices. Except for the nanoparticle size, the lifting speed, precursor concentration, and dipping number are the main parameters during the dip-coating processes. Herein, the lifting speed, precursor concentration, and dipping number were also varied to investigate their effects on the microstructure of TiO_2_ thin films as well as the performance of electrochromic devices, i.e., transmittance/reflectance, optical contrast, switching time, and cycling stability. The results in this study will provide valuable guidance for rational design of the electrochromic device with satisfactory performance.

## Methods

### Materials

FTO transparent conducting glasses with the size of 25 × 30 mm, the thickness of 2.2 mm, and a sheet resistance of 10 Ω sq^−1^ were used as the electrodes, which were purchased from Wuhan Lattice Solar Energy Technology Co. Ltd. Uniform TiO_2_ nanoparticles with average diameters of 5~10, 40, and 100 nm (Aladdin Co. Ltd.) were used to modify the FTO electrodes. Electrolyte compounds including dimethyl sulfoxide (DMSO, ≥99.8%, J&K Chemical Co. Ltd.), tetra-*n*-butylammoniumbromide (TBABr, ≥99%, J&K Chemical Co. Ltd.), silver nitrate (AgNO_3_, ≥99.8%, Guangdong Guanghua Sci-Tech Co. Ltd.), copper chloride (CuCl_2_, ≥99.0%, KeLong Chemical Co. Ltd.), poly (vinyl butyral) (PVB, Sekisui Chemical Co. Ltd.), ethyl cellulose (≥99.5%, Hanzhou Lanbo Industrial Co. Ltd.), lauric acid (≥99.8%, KeLong Chemical Co. Ltd.), terpineol (≥98.0%, KeLong Chemical Co. Ltd.), and ethyl alcohol (≥99.7%, KeLong Chemical Co. Ltd.) were obtained from commercial sources. All solvents and chemicals were of reagent quality and were used without further purification. Teflon sheets (Aladdin Co. Ltd.) with a thickness of 0.5 mm were cut to 25 × 25 mm with a 20 × 20 mm hole. Both FTO glass electrodes and Teflon sheets were cleaned with ethanol and de-ionized water several times before use.

### Preparation of TiO_2_ Nanoparticle Dispersion and Gel Electrolyte

To prepare the TiO_2_ nanoparticle dispersion, TiO_2_ nanoparticles (raw materials, 2.5 g) with lauric acid (surfactant, 0.25 g) and ethyl cellulose (adhesive, 0.75 g) were placed into a ball-mill jar at first and mixed with terpineol (adhesive, 16 mL) and ethyl alcohol (solvent, 10 mL) immediately before milling. TiO_2_ nanoparticle slurry was obtained after 50 min milling, followed by diluting the slurry with ethyl alcohol. To prepare the gel electrolyte, TBABr (806 mg, 2.5 mmol), silver nitrate (85 mg, 0.5 mmol), and copper chloride (13 mg, 0.1 mmol) were dissolved in 10 mL of DMSO, followed by the addition of PVB (1.32 g, 10 wt%). Finally, the mixed solution was placed in the dark for 24~48 h to obtain the gel electrolyte.

### Modification of FTO Electrode and Fabrication of Electrochromic Devices

Dip-coating technique was used to modify the FTO transparent conducting electrode, with a typical process as follows: ethyl alcohol (10, 15, or 20 mL) as a diluent was added to TiO_2_ nanoparticle dispersion (5 mL), ultrasonically mixed for 30 min. Subsequently, the FTO electrode with tap pasted on the whole back and the upper front was fixed on the dip coater, immersed into the aforementioned dispersion with speed of 6000 μm/s, and lifted with speeds of 1000, 2000, and 3000 μm/s, respectively. The TiO_2_ nanoparticle-modified FTO conducting electrode was obtained by sintering the as-prepared samples for 30 min at 500 °C. For comparison, TiO_2_ nanoparticles with different sizes (5~10, 40, and 100 nm) were used, and different parameters of dip-coating, including lifting speed (1000, 2000, and 3000 μm/s), precursor concentration (ratios of TiO_2_ nanoparticle dispersion and ethyl alcohol of 1:2, 1:3, and 1:4), and dipping number (1, 3, and 5) were used in this study. Specifically, to investigate the effects of TiO_2_ nanoparticle size on the performance of electrochromic device, TiO_2_ nanoparticles with sizes of 5~10, 40, and 100 nm were used by fixing the lifting speed to be 3000 μm/s, the precursor concentration to be 1:2, and the dipping number to be 1. To investigate the effects of lifting speed on the performance of electrochromic device, lifting speeds of 1000, 2000, and 3000 μm/s were used by fixing the TiO_2_ nanoparticle size of 5~10 nm, the precursor concentration to be 1:2, and the dipping number to be 1. To investigate the effects of precursor concentration on the performance of electrochromic device, ratios of TiO_2_ nanoparticle dispersion and ethyl alcohol of 1:2, 1:3, and 1:4 were used by fixing the TiO_2_ nanoparticle size of 5~10 nm, the lifting speed to be 3000 μm/s, and the dipping number to be 1. To investigate the effects of dipping number on the performance of electrochromic device, dipping numbers of 1, 3, and 5 were used by fixing the TiO_2_ nanoparticle size of 5~10 nm, the lifting speed to be 3000 μm/s, and the precursor concentration to be 1:2. To assemble the electrodeposition-based electrochromic device, DMSO-based gel electrolyte was contained in a hermetic square space of 20 mm × 20 mm, cut inside a 0.5-mm thick Teflon sheet, and sealed by sandwiching the Teflon sheet between two FTO electrodes (one of which was modified with TiO_2_ nanoparticles).

### Characterization

A field-emission scanning electron microscope (FESEM, S-3400, Hitachi) was used to observe the morphology of TiO_2_ nanoparticle-modified FTO electrodes. The roughness of TiO_2_ nanoparticle-modified FTO electrodes were characterized by using an atomic force microscope (AFM, Multimode V, Veeco). The transformation voltage was applied to the electrochromic devices using an electrochemical workstation (CHI660D, CHI), and the transmittance and reflectance spectra were measured using a UV-Vis spectrophotometer (Cary 5000, Agilent). All the electrochromic properties including optical contrast, switching time, and cycling stability were obtained by using a two-electrode mode, with the negative pole and positive pole connected to the flat FTO electrode and TiO_2_ nanoparticle-modified FTO electrode, respectively. The counter electrode of the electrochromic device during the measurement was the flat FTO electrode, and the working electrode was the TiO_2_ nanoparticle-modified FTO electrode. By applying suitable voltages, the dip-coated electrochromic device exhibited three reversible optical states, including transparent, mirror, and black.

## Results and Discussion

**Fig. 1:**
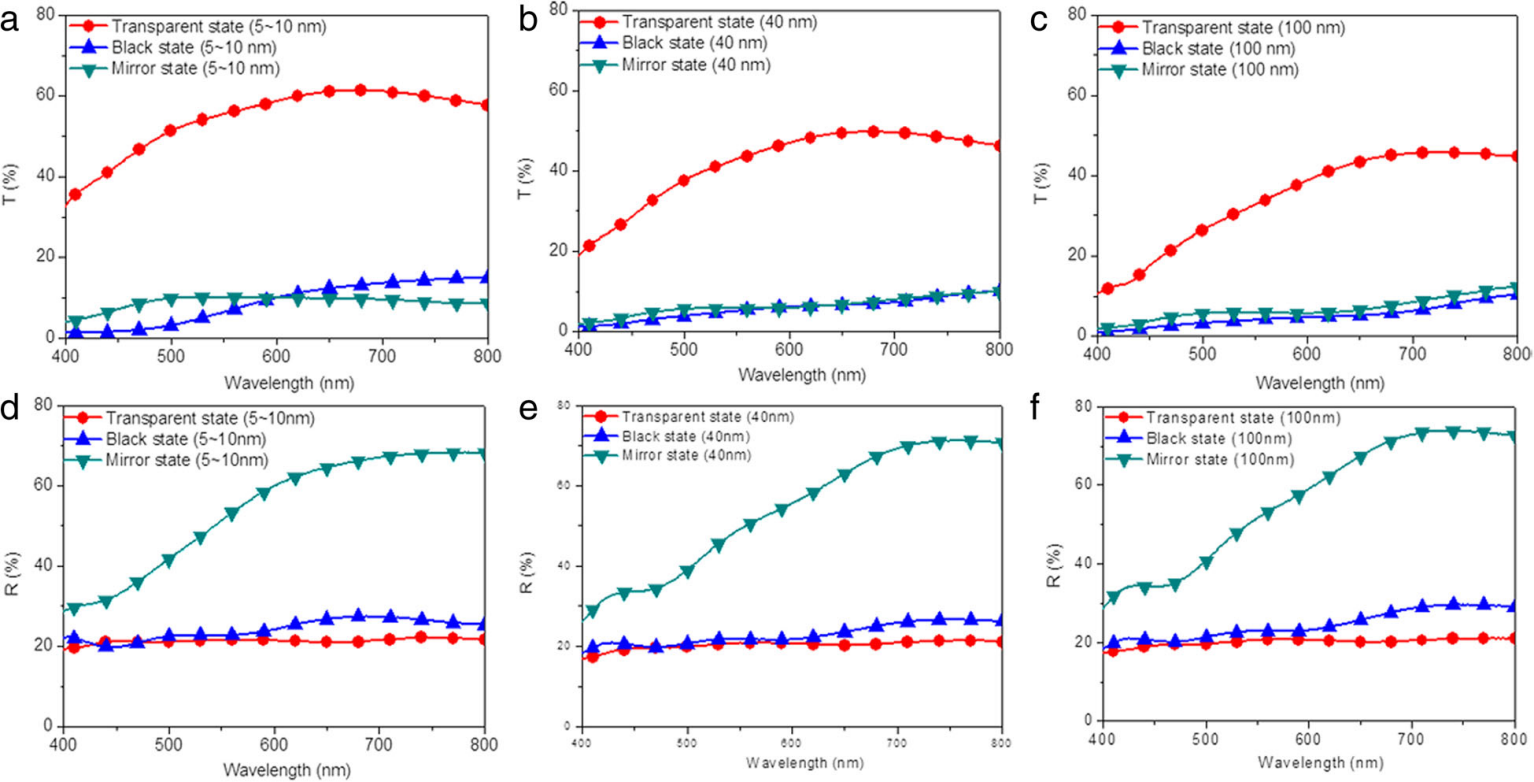
(Color online) Optical properties of the electrodeposition-based electrochromic device in transparent (*red*), black (*blue*), and mirror states (*green*). Transmittance spectra of modified devices prepared with **a** 5~10, **b** 40, and **c** 100 nm, respectively. Reflectance spectra of modified devices prepared with **d** 5~10, **e** 40, and **f** 100 nm, respectively

Reversible three-state optical transformation among mirror, black, and transparent states can be achieved by alternately applying/removing suitable voltages on the electrodeposition-based electrochromic device. The black and mirror states would be triggered for Ag deposited on rough TiO_2_ nanoparticle-modified FTO electrode and on the flat electrode, respectively. Accordingly, the black state of the modified device can be strongly influenced by their surface morphological structures. To investigate the effects of the surface morphological structure of the TiO_2_ thin film on the performance of the modified devices, three precursor solutions containing TiO_2_ nanoparticles with different sizes (5~10, 40, and 100 nm) were prepared by ball-milling. Subsequently, the modified devices were obtained by coating precursor solutions onto the surface of FTO electrodes via dip-coating technique, sintering treatments, and sandwiching a suitable amount of gel electrolyte between the modified FTO electrodes and the flat FTO electrodes. Firstly, the optical transmittance and reflectance spectra of the three modified devices in transparent, mirror, and black states were measured in the spectra region from 400 to 800 nm. For transmittance measurement, the negative pole and positive pole of a power source were connected to the flat FTO electrode and TiO_2_ nanoparticle-modified FTO electrode, respectively, resulting in mirror state with +2.5 V and black state with −2.5 V after 20 s. For reflectance measurement, the same voltages were applied for 90 s. In the transparent states, the transmittance of 61, 50, and 46% are observed for modified devices prepared with the TiO_2_ nanoparticles of 5~10, 40, and 100 nm, respectively (Fig. 1a–c). In the black states, the modified device prepared with the TiO_2_ nanoparticles of 5~10 nm shows the maximum transmittance of 15% and decreases to 10% when increasing the size of TiO_2_ nanoparticles to 100 nm (Fig. 1a–c). In the mirror states, the modified device prepared with the TiO_2_ nanoparticles of 5~10 nm shows similar transmittance with those of 40 and 100 nm (Fig. 1a–c). The optical contrast is usually defined as the maximal difference of transmittance, reflectance, or absorbance for an electrochromic device between its coloration and bleaching processes. By calculating the difference of transmittance for a device between transparent and black states, optical contrasts of 48, 42, and 39% are obtained. The decreased optical contrast with the increase of TiO_2_ nanoparticle size is mostly attributed to the decreased transmittance for the device in a transparent state. The reflectance peak of the modified device prepared with 5~10 nm TiO_2_ nanoparticles is different from that prepared with 40 and 100 nm TiO_2_ nanoparticles, with peak positions at 700, 750, and 750 nm, respectively (Fig. 1d–f). Basically, the refractive index is usually decided by materials, structure (i.e., the number and arrangement of the membranes), thickness, and interface morphology/structure of the membrane. Thus, the reason for this peak shift in the wavelength-dependent reflectance spectra of the modified electrodeposition-based electrochromic device in a mirror state may be the combined effects of the varied TiO_2_ nanoparticle size, the TiO_2_ thin film thickness, and the TiO_2_ thin film surface roughness [45–47]. Furthermore, reflectance over 70% are observed for modified devices in mirror states, with a low reflectance of 20% observed for the three modified devices in black and mirror states exhibited (Fig. 1d–f). It should be noted that the above values do not correspond to the darkest state that can be reached. The aforementioned results suggest that the optical transmittance, optical reflectance, and optical contrast of the electrodeposition-based device can be altered by the size of TiO_2_ nanoparticles that deposited on the transparent electrode.

The structural features of the dip-coated TiO_2_ thin films with different nanoparticle sizes were investigated. X-ray diffraction (XRD) patterns for the sintered dip-coated TiO_2_ films, as-prepared dip-coated TiO_2_ films, fresh TiO_2_ nanoparticles without further treatment, and bald FTO transparent conductive electrode are recorded in the 2*θ* range from 20° to 80° [33, 48]. As presented in Additional file 1: Figure S1a, the diffraction peaks of as-prepared TiO_2_ film, sintered TiO_2_ films, and fresh 5~10 nm TiO_2_ nanoparticles without further treatment occur at the same positions and match very well with anatase structural form of TiO_2_ (TiO_2_ anatase, JCPDS 21-1217). These values are in good agreement with literature data [48], with widened dispersion peaks appear in correspondence with crystal planes (101), (004), (200), (105), (211), and (204) of anatase phase. The observed extra peaks at 52° and 62° come from the FTO electrode surface, which matches well with the structural form of a tin oxide (SnO_2_, JCPDS 46-1088) [33, 49]. Similar anatase structural form are also observed for 40 and 100 nm TiO_2_ nanoparticle-modified FTO electrodes before and after sintering (Additional file 1: Figure S1b and S1c). It can be seen that the dip-coated TiO_2_ thin films remain the same structural form as fresh TiO_2_ nanoparticles throughout the whole fabricating procedure for modifying FTO electrode, indicating that the structural features of the coated TiO_2_ thin films will not be influenced by the dip-coating methods, with similar results also presented by our previous report [33].

**Fig. 2:**
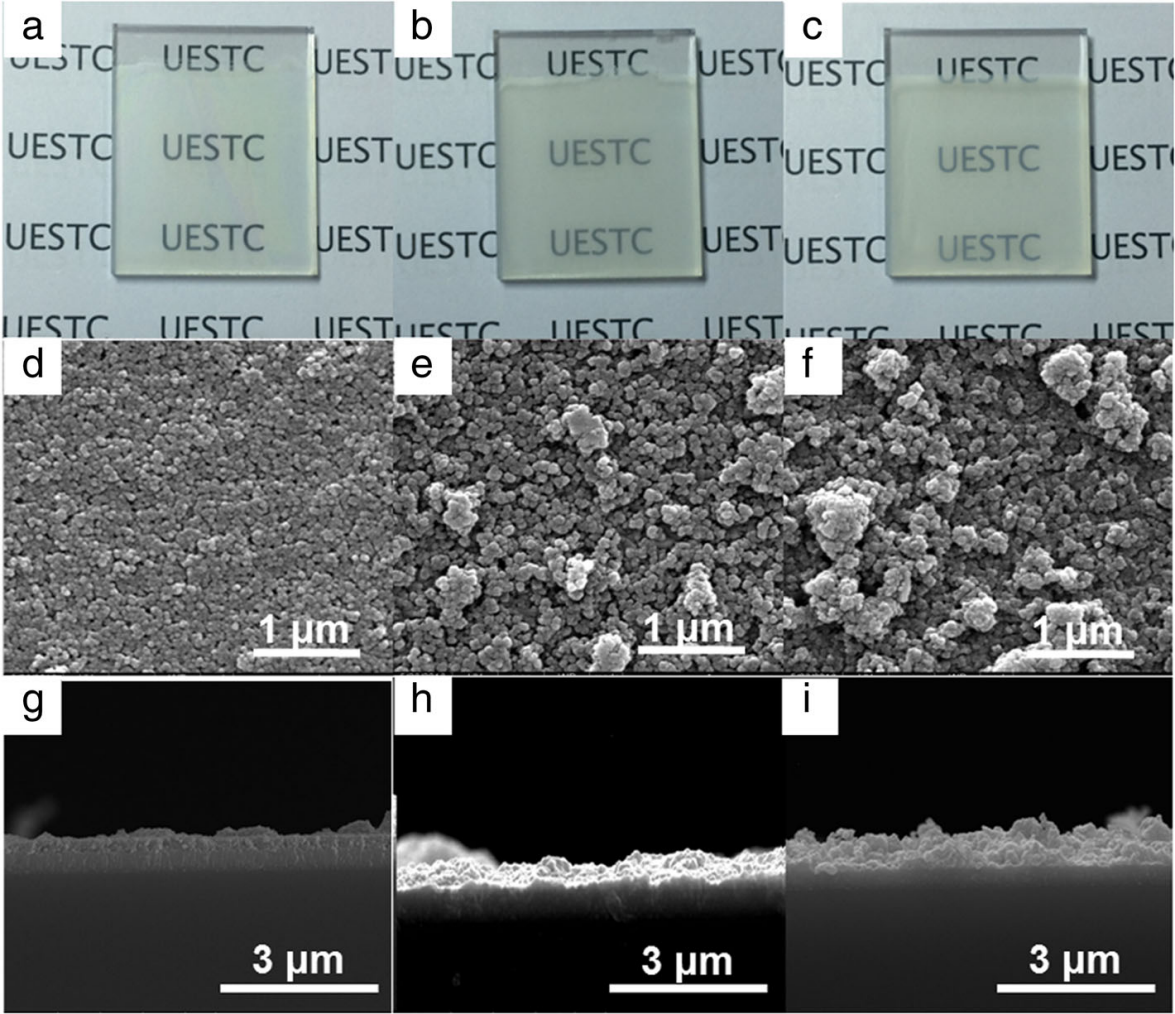
(Color online) Photographs of TiO_2_ thin films prepared with nanoparticle sizes of **a** 5~10, **b** 40, and **c** 100 nm, respectively. In-plane SEM images of TiO_2_ thin films prepared with nanoparticle sizes of **d** 5~10, **e** 40, and **f** 100 nm, respectively. Cross-sectional SEM images of TiO_2_ thin films prepared with nanoparticle sizes of **g** 5~10, **h** 40, and **i** 100 nm, respectively

Secondly, the morphological features of the three dip-coated TiO_2_ thin films were investigated. Photographs, in-plane and cross-sectional SEM images of the dip-coated TiO_2_ thin films before Ag deposition, were provided in Fig. 2. The FTO electrodes deposited with TiO_2_ nanoparticles show different transparency and gradually blurred after increasing the size of TiO_2_ nanoparticles (Fig. 2a–c). The thin film prepared with TiO_2_ nanoparticles of 5~10 nm shows sharp and well-defined boundaries between grains as well as uniform distribution of pores and grains, indicating a homogeneous and fine-grained TiO_2_ thin film obtained (Fig. 2d). After increasing the size of TiO_2_ nanoparticles, the surface of deposited TiO_2_ thin film, however, becomes rough and inhomogeneous (Fig. 2e, f). This inhomogeneous distribution of TiO_2_ nanoparticles mainly results from their gradual reduction of dispersity in ethyl alcohol and agglomeration during the dip-coating and sintering processes. The gradually blurred and rougher TiO_2_ thin film with increasing size of nanoparticles illustrates the decreased transmittance spectra for modified devices, as shown in Fig. 1a. Typically, an increase in thickness of the three TiO_2_ thin films are measured through the cross-sectional SEM images, with the thicknesses of TiO_2_ thin film of 320, 409, and 612 nm for FTO electrodes prepared with 5~10, 40, and 100 nm TiO_2_ nanoparticles observed, respectively. During dip-coating process, the continuous thin film can be obtained through the balance among particle gravity, lifting force, and capillary force during the solvent evaporation process. Different balance force, resulted from varied particle gravity and capillary force, is expected for different nanoparticle sizes, which leads to different thickness and roughness. As illustrated in Fig. 1a, b, the transmittance, reflectance, and optical contrast of the modified device in the transparent state are changed after increasing the TiO_2_ nanoparticle size. It thus can be seen that the lowered optical transmittance of the modified electrochromic device in the transparent state for the enlarged TiO_2_ nanoparticle size can be mainly explained by the increased thickness of the dip-coated TiO_2_ thin films.

**Fig. 3:**
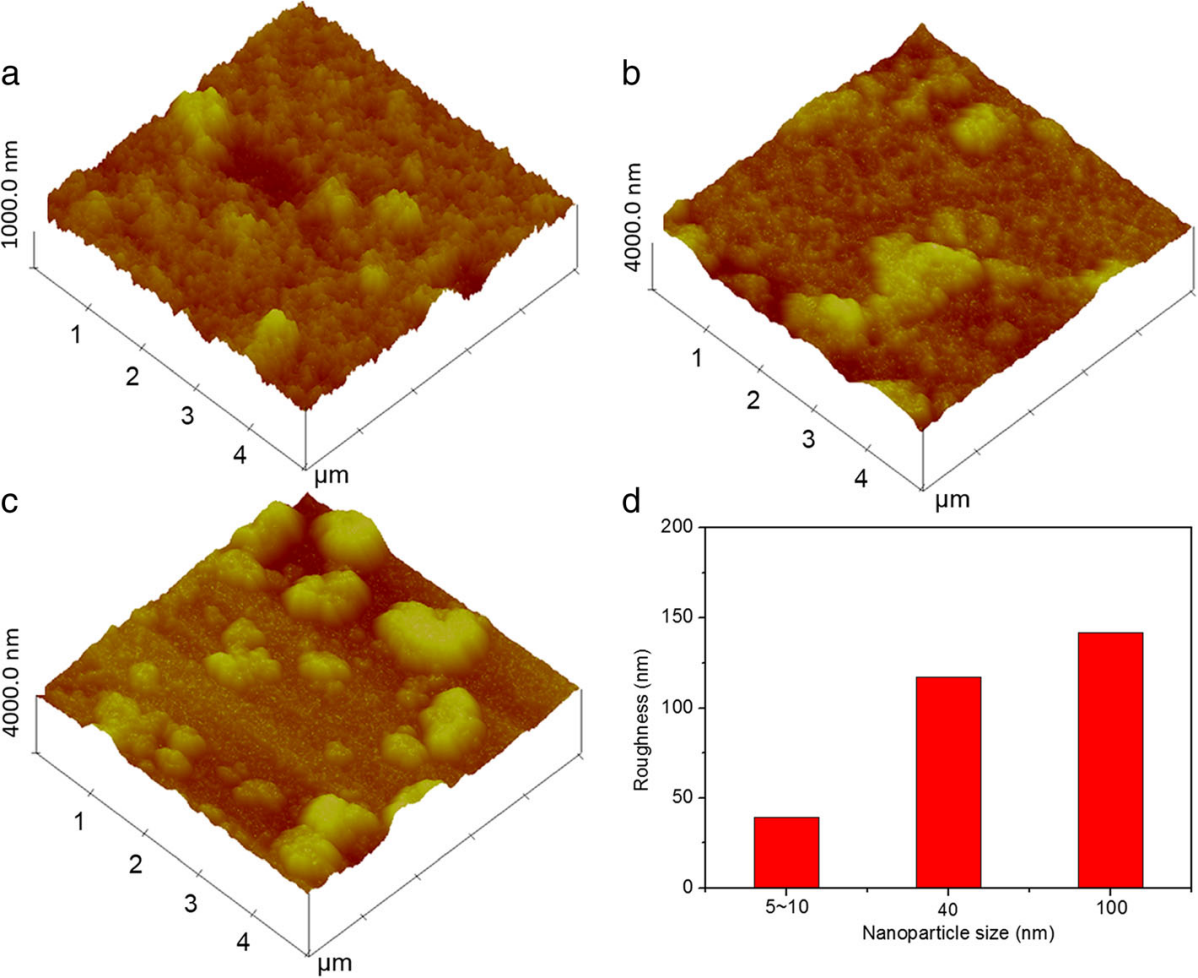
(Color online) AFM images of TiO_2_ thin film prepared with nanoparticle sizes of **a** 5~10, **b** 40, and **c** 100 nm and **d** roughness of TiO_2_ thin film as a function of TiO_2_ nanoparticle size

The roughness of the three dip-coated TiO_2_ thin films was further measured by using an atomic force microscope (AFM), as shown in Fig. 3a–c. The roughness of the dip-coated TiO_2_ thin films as a function of nanoparticle size was plotted in Fig. 3d, with the roughness of 39, 117, and 142 nm for TiO_2_ thin films prepared with 5~10, 40, and 100 nm TiO_2_ nanoparticles measured, respectively. The increase in roughness is observed as a result of more aggregation and lower dispersity for larger TiO_2_ nanoparticles. Basically, transmittance and related reflectance are used to describe the behavior of wave incident to devices. Refractive index factor, an essential indicator, is decided by materials, structure (i.e., the number and arrangement of the membranes), thickness, and interface morphology/structure of the membrane. All the aforementioned factors should be taken into account when investigating the optical properties of the modified electrodeposition-based electrochromic devices with different sizes of TiO_2_ nanoparticles. After triggering the black states of modified devices, all the FTO surfaces turned dark black, indicating that the Ag layers modify the surface morphology of the FTO electrodes significantly (Additional file 1: Figure S2a, S2b, and S2c). All the FTO surfaces of modified devices with deposited Ag layer get smoother than those coated with bald TiO_2_ thin films (Additional file 1: Figure S2d, S2e, and S2f). The cross-sectional SEM images of the dip-coated TiO_2_ thin films (Additional file 1: Figure S2g, S2h, and S2i) also exhibit thick and compact Ag deposited layers for all the three modified devices. As shown in Fig. 1, transmittance and reflectance spectra are altered after Ag deposition and the devices transform to the black states, indicating a strong influence of thickness and roughness on transmittance and reflectance. The combined effects of the changed membrane structure, including the additional deposited Ag layer, the altered thickness, and the interface morphology of the top layer, should be considered.

**Fig. 4:**
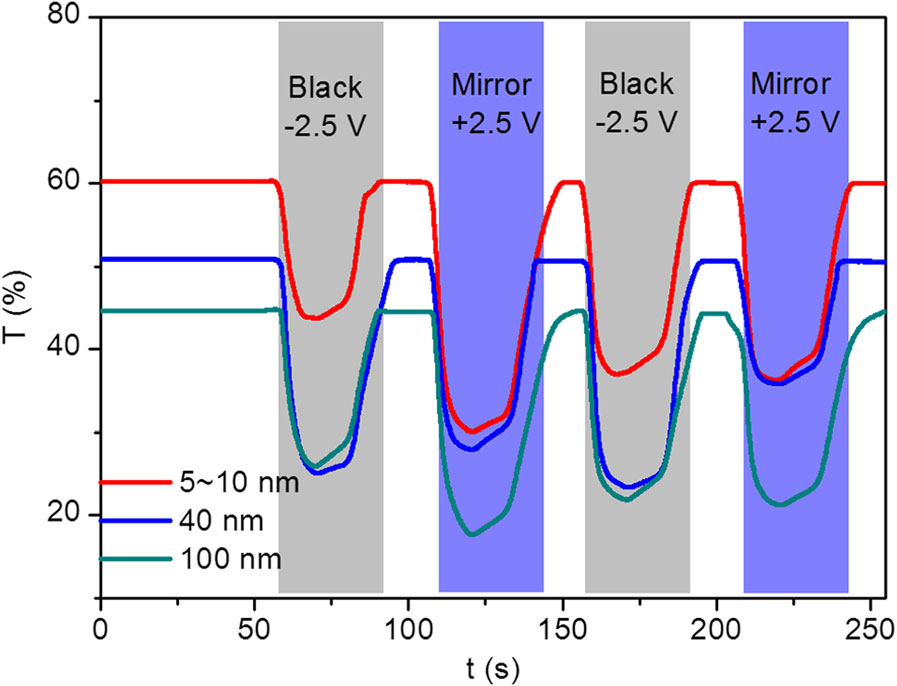
(Color online) Transmittance variations at 700 nm obtained during two-electrode CV tests for devices modified with TiO_2_ nanoparticle sizes of 5~10 (*red*), 40 (*blue*), and 100 nm (*green*), respectively

Thirdly, time-dependent transmittance changes of the three modified devices at 700 nm were measured during two-electrode cyclic voltammogram (CV) tests, with four consecutive coloration/bleaching cycles and a sweep rate of 100 mV/s. For transmittance measurement, voltages of +2.5 and −2.5 V were alternately applied to the TiO_2_-modified FTO electrodes for 20 s. Figure 4 shows the transmittance variation over time for modified devices prepared with TiO_2_ nanoparticles of different sizes. The initial transmittance of the modified devices prepared with 5~10, 40, and 100 nm TiO_2_ nanoparticles attain 61, 50, 46% upon bleaching and drops to 34, 25, 18% upon coloration, respectively. Basically, the coloration process means the device changes from transparent state to mirror/black state, and the bleaching process means the device reversely changes from mirror/black state to transparent state. Coloration/bleaching switching time is expressed as the time needed to reach 90% of its maximum modulation during coloration and bleaching processes. Different switching times were measured for devices modified with TiO_2_ nanoparticles of different sizes, with the modified device prepared with 5~10 nm TiO_2_ nanoparticles exhibiting the shortest switching time (6 s for coloration and 20 s for bleaching) between coloration and bleaching processes. The increased switching time with the increase of nanoparticle size illustrates that an FTO electrode modified with thinner and smoother TiO_2_ thin film contributes to the shorter coloration/bleaching switching time. Furthermore, the bleaching process is slower than the coloration process for all devices, which is illustrated by most articles about electrochromic devices. Moreover, the time for modified devices to transform from transparent to mirror states is shorter than that for the device from transparent to the black state, indicating that the rough TiO_2_ thin films deposited on the FTO electrodes will influence their switching time. Furthermore, recent developments in the processing of porous transition metal oxide thin films have opened up new opportunities in the construction of electrochromic devices with enhanced properties. For example, Zhang et.al reported that electrodeposited periodical bowl-like macroporous WO_3_ array film electrodeposited on ITO glasses by using self-assembled monolayer polystyrene (PS) spheres as template show a much faster coloration time of 3.6 s, when compared with dense film prepared without PS template [50]. Yang and co-workers reported the fabrication of ordered macroporous WO_3_ thin films prepared via template-assisted sol–gel method. The coloration time is obtained to be 5.19 s, which is noticeably shorter than that of dense films, namely, 6.9 s [51]. They also demonstrated that the electrochromic response time is actually limited by two factors: the ion diffusion coefficient and the length of diffusion path, with the former one depends on the chemical structure, while the latter depends on the microstructure.

The coloration efficiency, CE (cm^2^/*C*), is one of the best parameters often used to evaluate an electrochromic device. CE is defined as the change in the optical density (ΔOD) per unit of injected/extracted charge (*Q*) at a certain wavelength [17], which can be calculated from the following formula

$$ \mathrm{C}\mathrm{E}\left(\lambda \right)=\Delta \mathrm{O}\mathrm{D}\left(\lambda \right)/ Q = \log \left( T\mathrm{b}/ T\mathrm{c}\right)/ Q $$

where ΔOD is the change in the optical density, *Q* (*C*/cm) is the charge injected per unit electrode area of the thin film, and *T*_b_ and *T*_c_ are the transmittance in the bleached and the colored states, respectively. The coloration efficiency of the modified devices prepared with 5~10, 40, and 100 nm TiO_2_ nanoparticles were listed in Additional file 1: Table S1. CE of 27.0, 20.7, and 16.9 cm^2^/C at 700 nm were obtained for modified devices prepared with 5~10, 40, and 100 nm TiO_2_ nanoparticles, respectively. The decreased CE value indicates that the modified devices prepared with 5~10 nm exhibits a large optical modulation with a small intercalation charge density. This decreased CE of the electrochromic devices may be due to the increased TiO_2_ nanoparticle size and TiO_2_ thin film thickness and roughness, as demonstrated by previous reports [52–54].

**Fig. 5:**
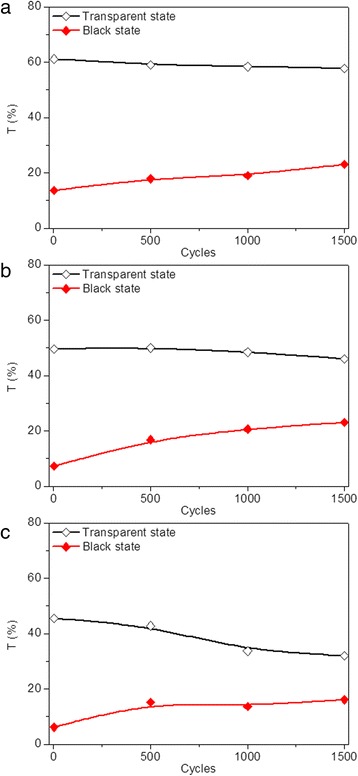
(Color online) Transmittance variation for dip-coated devices prepared with TiO_2_ nanoparticles of **a** 5–10, **b** 40, and **c** 100 nm, respectively, in transparent (*black*) and black (*red*) states at 700 nm after applying a sequence of voltages in the following order: −2.5 V (10 s), 0.5 V (30 s), 2.5 V (10 s), 0.5 V and (20 s), with each of the 500 cycles taken as a measurement node

Generally, device failure occurs after repeatedly switching an electrochromic device between its coloration and bleached states for hundreds or thousands of times. This attributes to the combined effect of various side reactions including transparent electrode failure, electrolyte depravation, and active layer decay. Thus, cycling stability of the modified devices is further investigated by repeatedly applying sequential voltages. As shown in Fig. 5, transmittance variation of the three modified devices at 700 nm was measured by applying voltages of −2.5 V. Every 500 cycles was taken as a measurement node to measure the transmittance deviation of modified devices over time. The measured transmittance of the three devices are all below 1% and maintain fairly stable after the devices transferred into black state and the voltage removed for the first cycle. The transmittance of the modified devices in transparent states gradually decrease and increase for coloration states with the time and the cycle numbers, indicating more cycles lead to poorer stability. The optical contrast of the modified device prepared with 5–10 nm TiO_2_ nanoparticles decreases from 48 to 35% after 1500 cycles (Fig. 5a). As shown in Fig. 5b, c, the optical contrasts of modified devices prepared with 40 and 100 nm TiO_2_ nanoparticles decrease to 23 and 16%, respectively, indicating that the cycling stability can be improved by decreasing the size of TiO_2_ nanoparticles. To investigate the trace of Ag dissolution in the electrolyte for the sample with less stability, the morphological features of the dip-coated TiO_2_ thin film after manifold cycles were investigated. The SEM of dip-coated 100 nm TiO_2_ thin film after 1500 cycles was presented in Additional file 1: Figure S3. As shown in Additional file 1: Figure S3, uneven electrolyte agglomeration is observed for the dip-coated TiO_2_ thin film in our work, which is similar to that of the previous reports [55, 56]. Moreover, extra Ag was detected via energy-dispersive spectrometer (EDS) in the dip-coated TiO_2_ thin film after the transformation of modified device to the transparent state, with the results listed in the Additional file 1: Table S2. We supposed that the ability to achieve complete reversibility is deteriorated due to the gradual deposition of Ag onto bumps of TiO_2_ thin films and inability to dissolve Ag back into electrolyte immediately during the continuous cycling between the coloration and bleaching states. Thus, the improved cycling stability might be owed to the decreased surface roughness of TiO_2_ thin film, which is conducive to the quick dissolution of Ag back into electrolyte during the switching between the coloration and bleaching states, and the surface roughness of TiO_2_ thin film is strongly influenced by the TiO_2_ nanoparticle size. Therefore, both the particle size itself and the surface roughness are related to the improved cycling stability.

**Fig. 6:**
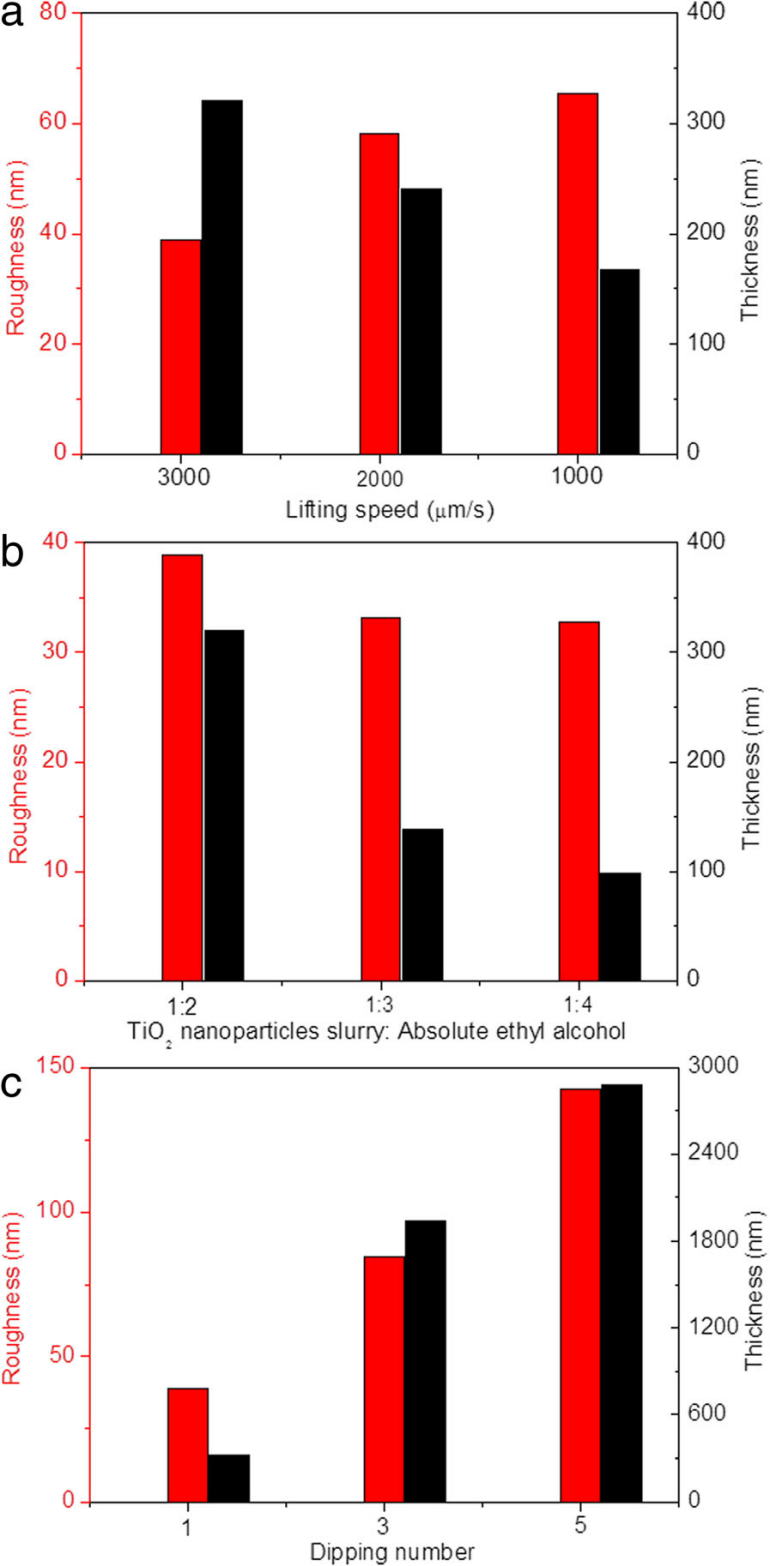
(Color online) Roughness (*red*) and thickness (*black*) of TiO_2_ thin films prepared with **a** different lifting speeds, **b** precursor concentrations, and **c** dipping numbers provided

In summary, improved optical contrast, switching time, and cycling stability were obtained with the decrease of TiO_2_ nanoparticle size, indicating that the effect of nanoparticle size on the electrochromic device is obvious in this work. By characterizing the SEM and AFM images of different-sized TiO_2_ thin film, increased thickness and roughness of the dip-coated TiO_2_ thin film are exhibited with the increase of TiO_2_ nanoparticle size, which results to the varied properties of electrochromic device, indicating the strong relevance between the TiO_2_ nanoparticle size and the morphological feature of the dip-coated TiO_2_ thin films. To effectively distinguish the effect of TiO_2_ nanoparticle size and TiO_2_ thin film morphological feature on the properties of modified electrochromic device, TiO_2_ thin films were deposited onto FTO electrodes under different modification conditions, including lifting speed, precursor concentration, and dipping number, by fixing the TiO_2_ nanoparticle size to 5~10 nm. The thickness and roughness of TiO_2_ thin films prepared with different lifting speeds, precursor concentrations, and dipping numbers were plotted in Fig. 6. To compare the effects of different lifting speeds, lifting speeds of 3000, 2000, and 1000 μm/s were used to deposit TiO_2_ nanoparticles onto the FTO electrodes, with nanoparticle size of 5~10 nm, ratios between TiO_2_ nanoparticle slurry and absolute ethyl alcohol of 1:2, and dipping number of 1. Figure 6a shows that the increase of lifting speed leads to the increased thickness and decreased roughness of the modified electrodes. To compare the effects of different precursor concentrations, ratios between TiO_2_ nanoparticle slurry and absolute ethyl alcohol of 1:2, 1:3, and 1:4 were used to modify the FTO electrodes, with nanoparticle size of 5~10 nm, lifting speed of 3000 μm/s, and dipping number of 1. The result in Fig. 6b reveals that the decrease of precursor concentration causes the decreased thickness and roughness of the modified electrodes. To compare the effects of different dipping numbers, dipping numbers of 1, 3, and 5 were used to prepare the modified electrode, with nanoparticle size of 5~10 nm, lifting speed of 3000 μm/s, and ratios between TiO_2_ nanoparticle slurry and absolute ethyl alcohol of 1:2. The increase in both roughness and thickness are observed with the increase of dipping number, as indicated in Fig. 6c.

**Fig. 7:**
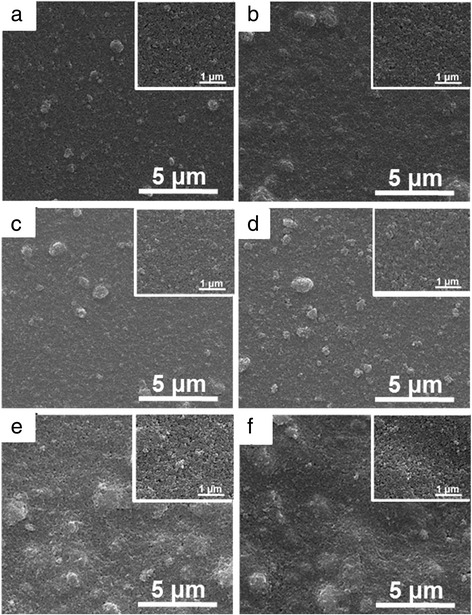
(Color online) SEM images of modified FTO electrodes under different fabrication conditions, including **a** lifting speed of 2000 μm/s, **b** lifting speed of 1000 μm/s, **c** precursor concentration of 1:3, **d** precursor concentration of 1:4, **e** dipping number of 3, and **f** dipping number of 5

In addition, the morphological features of dip-coated TiO_2_ thin films on modified electrodes prepared under different electrode modification conditions before Ag deposition were observed. Figure 7 shows the in-plane SEM images of modified FTO electrodes under different modification conditions, including lifting speed, precursor concentration, and dipping number. Compared with SEM image of TiO_2_ thin film dip-coated with 3000 μm/s, more agglomeration of TiO_2_ nanoparticles are observed for TiO_2_ thin films prepared under lower lifting speed (Fig. 7a, b). The increase in agglomeration of TiO_2_ nanoparticles leads to the increased roughness for lower lifting speed, as illustrated in Fig. 6a. SEM images with higher magnification are inserted in the upper-right corner for each low-magnification SEM image. Both the TiO_2_ thin films prepared with 2000 and 1000 μm/s show uniform distribution of pores and grains with sharp and well-defined boundaries between grains (Fig. 7a, b). As shown in Fig. 7c, d, slight agglomeration of TiO_2_ nanoparticles are also observed for TiO_2_ thin films with lower precursor concentration and with same roughness as that prepared with 3000 μm/s obtained (Fig. 2a). Furthermore, the higher magnification SEM images for TiO_2_ thin films prepared under lower precursor concentration also show compact TiO_2_ thin film surfaces. Moreover, the SEM images of TiO_2_ thin films prepared with different dipping numbers are also presented in Fig. 7e, f, with a large amount of TiO_2_ nanoparticle agglomeration observed at higher magnification. A lot of pores are exhibited for TiO_2_ thin films prepared by repeating dipping number, with more repeating times lead to more pores. Thus, it can be seen that the effects of nanoparticle size, lifting speed, precursor concentration, and dipping number on the roughness of dip-coated TiO_2_ thin films are different, which gets us thinking about that both the dispersity in ethyl alcohol and dip-coating processes will influence the resulted roughness. Therefore, it is essential to investigate the effects of the process engineering on morphological features of dip-coated TiO_2_ thin films. As aforementioned, there are strong correlations between optical properties of modified device and morphological features of dip-coated TiO_2_ thin films. Therefore, the optical properties for the electrodeposition-based electrochromic device, including transmittance and reflectance spectra, optical contrast, switching time, and cycling stability, should be further investigated.

**Fig. 8:**
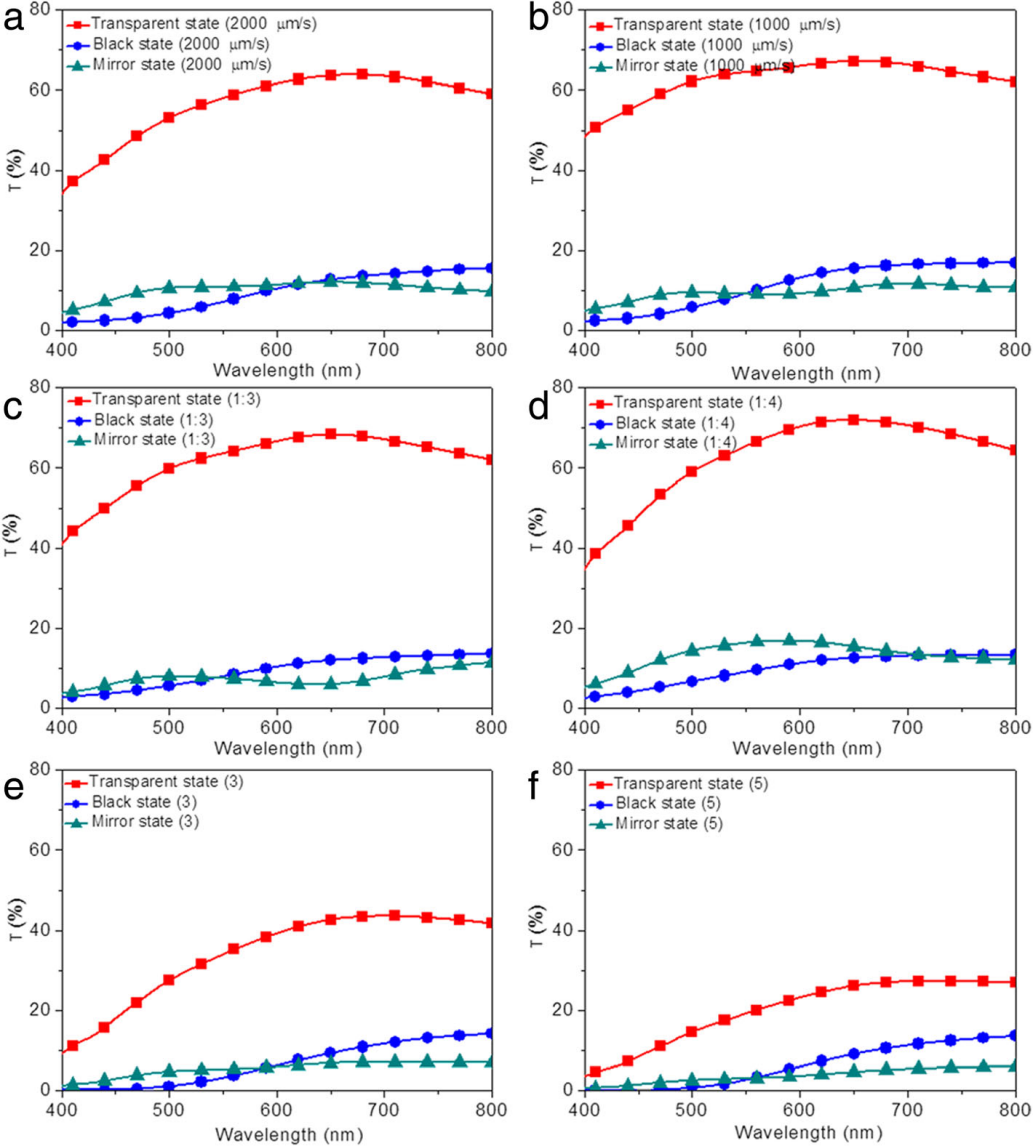
(Color online) Optical properties of the electrodeposition-based electrochromic device in transparent (*red*), black (*blue*), and mirror states (*green*). Transmittance spectra of modified devices prepared with different fabrication conditions, including **a** lifting speed of 2000 μm/s, **b** lifting speed of 1000 μm/s, **c** precursor concentration of 1:3, **d** precursor concentration of 1:4, **e** dipping number of 3, and **f** dipping number of 5

Optical transmittance of devices modified under different electrode modification conditions in three states were measured in the spectra range of 400 to 800 nm, as shown in Fig. 8. For modified devices prepared with different lifting speeds (2000 and 1000 μm/s), the transmittance of the device in the transparent state was decreased with increased lifting speed, as a result of the increased thickness of TiO_2_ thin film (Fig. 8a, b). As for the black and mirror states, limited variations are observed for modified devices prepared with 2000, 1000, and 3000 μm/s, as illustrated in Figs. 1a and 8a, b. Similarly, the measured transmittance does not correspond to the darkest state that can be reached. The modified devices prepared with 2000 and 1000 μm/s show the optical contrasts of 49 and 50%, respectively, which is slightly higher than that of the modified device prepared with 3000 μm/s (48%). For devices modified under different precursor concentration, increased transmittance of the modified devices in transparent states are obtained by decreasing the precursor concentration (Figs. 1a and 8c, d) for the combined effects of decreased thickness and roughness. Basically, the maximum transmittance of 70% is achieved for modified device prepared with precursor concentration of 1:4 (Fig. 8d). Similarly, both in the black and mirror states, all the modified devices prepared with different precursor concentrations show low transmittance, as illustrated in Figs. 1a and 8c, d. Optical contrasts of 54 and 57% are measured for modified devices prepared with precursor concentrations of 1:3 and 1:4, indicating increased optical contrast of modified device with decreased precursor concentration, which is attributed to the decreased thickness of TiO_2_ film. Moreover, decreased transmittance for devices modified with more dipping numbers are exhibited in Figs. 8e, f, which can be attributed to the increased thickness and roughness. The lowest transmittance of 27% is achieved by modified device prepared under the dipping number of 5. When the modified devices transform to black states, decreased transmittance (15, 14, and 13% for dipping number of 1, 3, and 5, respectively) are observed (Figs. 1a, f and 8e). As for the mirror states, same variation tendency for the three devices are observed, with the lowest transmittance of 5% achieved for the device by repeating the dip-coating process for five times (Fig. 8f). In addition, reflectance spectra for modified devices prepared with different electrode conditions are also illustrated in Additional file 1: Figure S4. In the transparent states, all the modified devices exhibit a low reflectance of ~20%. In the black states, the reflectance at 700 nm decreases from 33 to 25% upon increasing lifting speed from 1000 to 3000 μm/s (Additional file 1: Figure S4a and S4b). However, the influence of precursor concentration and dipping number on the reflectance of modified devices in black states is limited (Additional file 1: Figure S4c, S4d, S4e, and S4f). As for the mirror states, the same variation tendency for the modified devices is observed, with the highest reflectance over 80% achieved for the device by repeating the dip-coating process for five times (Additional file 1: Figure S4f). The aforementioned results indicate that the optical transmittance and reflectance of modified devices are strongly influenced by the electrode modification conditions. Furthermore, compared with the effects of TiO_2_ nanoparticle size and dipping number on the optical contrast of the electrodeposition-based devices, the effects caused by altering lifting speed and precursor concentration are not obvious, which is consistent with their different influence on thickness and roughness of TiO_2_ thin films.

**Fig. 9:**
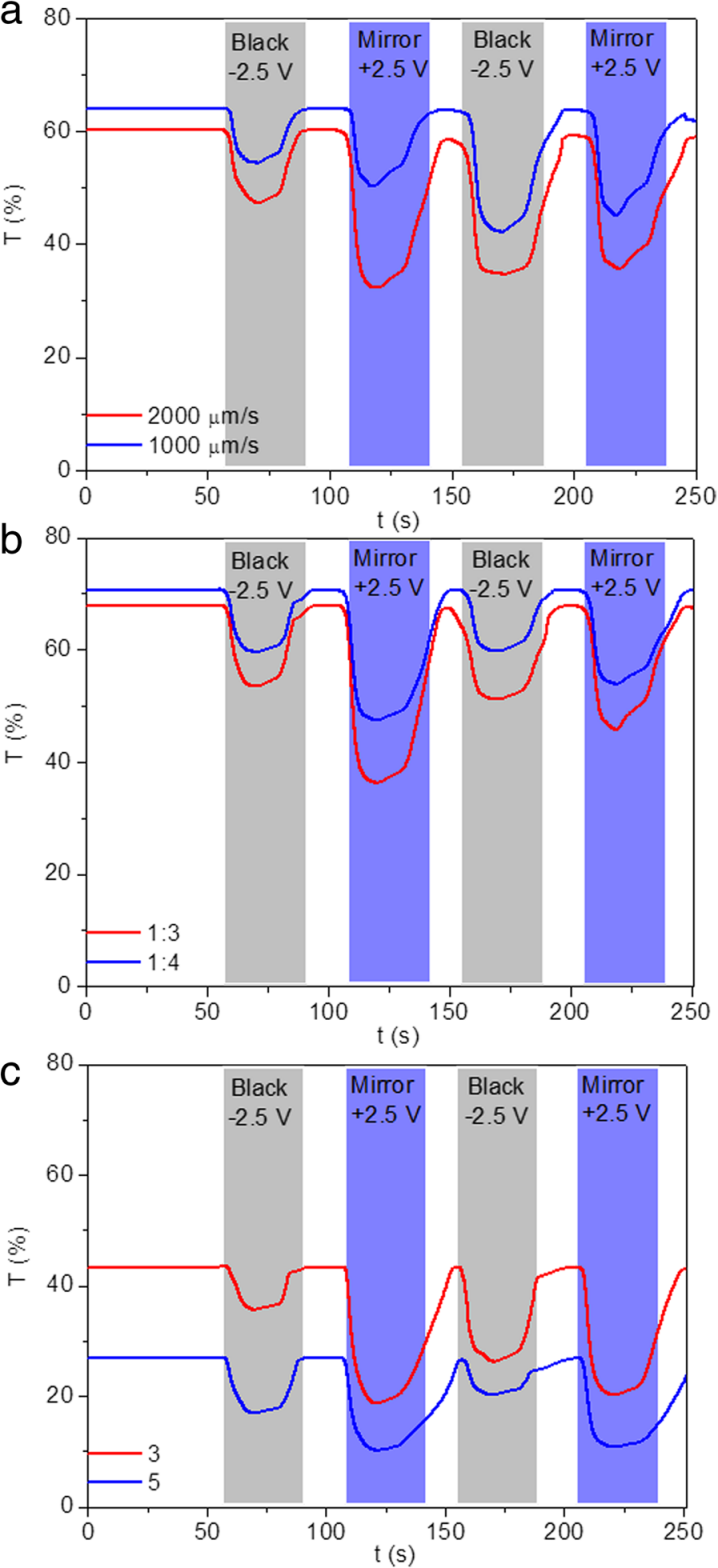
(Color online) Transmittance variation of TiO_2_ nanoparticles modified devices prepared under different modification conditions at 700 nm during two-electrode CV tests, including **a** different lifting speeds, **b** different precursor concentrations, and **c** different dipping numbers

As aforementioned, the switching time of the modified devices is strongly influenced by TiO_2_ nanoparticle size (Fig. 4). The transmittance of modified devices prepared under different electrode modification conditions in different optical states at 700 nm over time were measured to evaluate the corresponding switching time. As shown in Fig. 9a, longer switching times between coloration and bleaching states are observed for the modified devices prepared under lower lifting speeds. Furthermore, switching time for bleaching transition is slower than that for reverse transition, as illustrated in Fig. 4. For devices modified under different precursor concentrations, the switching speed is slowed down by reducing the ratios between TiO_2_ nanoparticle slurry and absolute ethyl alcohol, with coloration and bleaching time of 8 s for coloration and 30 s for bleaching measured (Fig. 9b). Similarly, longer switching time is observed for the devices with modified FTO electrodes prepared by repeating dipping number, with more dipping numbers leading to longer switching time (Fig. 9c). All the above results indicate that switching time of TiO_2_ nanoparticle-modified devices is strongly influenced by the modification conditions. Furthermore, considering the thickness and roughness of these dip-coated TiO_2_ thin films, the switching time of the modified devices can be accelerated by reducing TiO_2_ nanoparticle size and dipping number and increasing the lifting speed and precursor concentration. The coloration efficiency of the modified devices prepared with different fabrication parameters were also listed in Additional file 1: Table S1. Highest CE of 34 cm^2^/C is obtained for modified devices prepared with precursor concentration of 1:4, indicating the largest optical modulation with a small intercalation charge density.

**Fig. 10:**
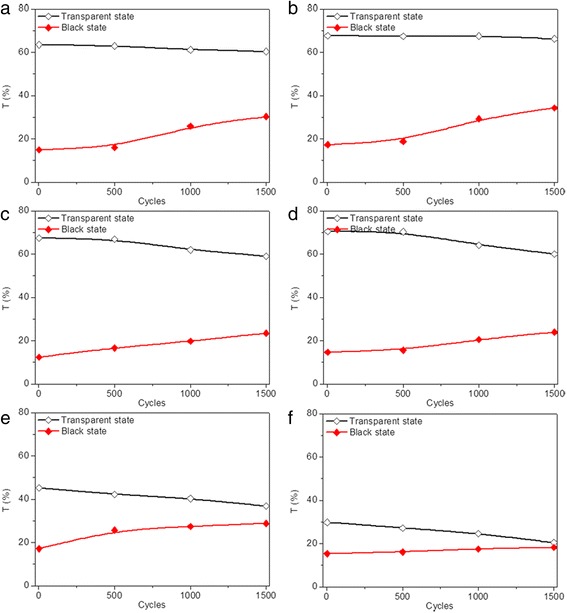
(Color online) Transmittance variation for dip-coated devices in transparent (*black*) and black (*red*) states prepared with different modification conditions at 700 nm during two-electrode CV tests, including **a**, **b** different lifting speeds, **c**, **d** different precursor concentrations, and **e**, **f** different dipping numbers after applying sequential voltages in the following order: 2.5 V (10 s), 0.5 V (30 s), 2.5 V (10 s), and 0.5 V (20 s), with each of the 500 cycles taken as a measurement node

The cycling stability of modified devices prepared under different electrode modification conditions were also evaluated by repeatedly applying sequential voltages. The transmittance of the modified devices, each after 500 cycles of state switching, is measured and plotted as a function of cycle numbers in Fig. 10. Similarly, all the modified devices exhibit transmittance below 1% and maintain fairly stable after the devices transfer into black state and the voltage removed for the first cycle, indicating excellent optical contrast. Decreased transmittance for devices in transparent states and increased transmittance for devices in coloration states are observed by increasing the cycle number. The decrement rate of optical transmittance contrast after 1500 cycles is measured to be 27, 36, and 40% for modified devices prepared with lifting speed of 3000 μm/s (Fig. 5a), 2000 μm/s (Fig. 10a), and 1000 μm/s (Fig. 10b), respectively, indicating ~33% improvement with the increase of lifting speed. The improved cycling stability owes to the decreased roughness of deposited TiO_2_ thin film with the increase of lifting speed, as illustrated in Fig. 4a. The decrement rates of optical contrasts of 33 and 37% are obtained after 1500 cycles for modified device prepared with precursor concentrations of 1:3 and 1:4, respectively (Fig. 10c, d), which are higher than those prepared with precursor concentration of 1:2 (Fig. 5a). The deteriorated cycling stability is mainly attributed to the dramatically increased transmittance for the device in a black state, which might be caused by the decreased thickness of TiO_2_ thin film on FTO electrode. Improved cycling stability is also achieved by reducing the dipping number (Fig. 10e, f).

## Conclusions

In summary, multi-state electrodeposition-based electrochromic devices with reversible three-state optical transformation were successfully prepared via a facile and well-controlled dip-coating technique. A systematic study of the correlation between dip-coating process engineering and the morphological features of the TiO_2_ nanoparticle-modified FTO electrodes as well as the optical behavior of the fabricated devices reveals that the performance of the three-state electrochromic device can be adjusted by simply manipulating the TiO_2_ nanoparticle size, lifting speed, precursor concentration, and dipping number. The optical properties of the assembled electrodeposition-based electrochromic devices, i.e., optical contrast, switching time, and cycling stability, strong depend on the thickness and roughness of the deposited TiO_2_ thin films, which are heavily influenced by the dip-coating process engineering. The high controllability of dip-coating technique and the obtained correlation between dip-coating process engineering and the morphological feature of the TiO_2_ nanoparticle-modified FTO electrodes as well as the optical performance of the fabricated devices provide valuable guidance for rational design and performance optimization of the electrochromic device with required optical properties. For the modified devices, the optical contrast of 57%, the coloration/bleaching switching time of 6 and 20 s, and the satisfactory cycling stability for the device after 1500 cycles are achieved by adjusting electrode surface modification. The TiO_2_ nanoparticle-modified device with reversible three-state optical transformation may have various applications, such as information displays and light-modulating devices.

## Additional file

Additional file 1:Figure S1. (Color online) XRD patterns of dip-coated TiO_2_ thin film (dip-coated on the FTO electrode and sintered at 500 °C for 30 min), spin-coated TiO_2_ thin film (spin-coated on the FTO electrode and sintered at 500 °C for 30 min), fresh TiO_2_ nanoparticles (purchased and untreated), flat FTO electrode (cleaned and dried). (a) 5~10, (b) 40, and (c) 100 nm, respectively. Figure S2. (Color online) Photographs of TiO_2_ thin films after Ag deposition with nanoparticle sizes of (a) 5~10, (b) 40, and (c) 100 nm, respectively. In-plane SEM images of TiO_2_ thin films after Ag deposition with nanoparticle sizes of (d) 5~10, (e) 40, and (f) 100 nm, respectively. Cross-sectional SEM images of TiO_2_ thin films after Ag deposition with nanoparticle sizes of (g) 5~10, (h) 40, and (i) 100 nm, respectively. Figure S3. (Color online) SEM images of dip-coated TiO_2_ thin film after switching between its coloration and bleached states for 1500 cycles. Figure S4. (Color online) Optical properties of the electrodeposition-based electrochromic device in transparent (red), black (blue), and mirror states (green). Reflectance spectra of modified devices prepared with different fabrication conditions, including (a) lifting speed of 2000 μm/s, (b) lifting speed of 1000 μm/s, (c) precursor concentration of 1:3, (d) precursor concentration of 1:4, (e) dipping number of 3, and (f) dipping number of 5. Table S1. Coloration efficiency (CE) of the electrochromic devices modified with different modification parameters. Table S2. Element ratios of TiO_2_ thin film prepared with 100 nm TiO_2_ nanoparticle after 1500 cycles. (DOCX 7367 kb)

## Abbreviations

AFM: Atomic force microscope
Ag: Silver
AgNO_3_: Silver nitrate
Bi: Bismuth
CE: Coloration efficiency
Cu: Copper
CuCl_2_: Copper chloride
CV: Cyclic voltammograms
DMSO: Dimethyl sulfoxide
EDS: Energy-dispersive spectrometer
FESEM: Field-emission scanning electron microscope
FTO: Fluorine-doped tin oxide
ITO: Indium tin oxide
Ni: Nickel
Pb: Plumbum
PVB: Poly (vinyl butyral)
RH: Relative humidity
TBABr: tetra-*n*-Butylammoniumbromide
TiO_2_: Titanium dioxide
WO_3_: Tungsten trioxide
XRD: X-ray diffraction

## References

[1] Deb SK (1969). A novel electrophotographic system. Appl Optics.

[2] Lu YR, Wu TZ, Chen CL, Wei DH, Chen JL, Chou WC, Dong CL (2015). Mechanism of electrochemical deposition and coloration of electrochromic V2O5 nano thin films: an in situ X-ray spectroscopy study. Nanoscale Res Lett.

[3] DeLongchamp DM, Hammond PT (2004). High-contrast electrochromism and controllable dissolution of assembled Prussian blue/polymer nanocomposites. Adv Funct Mater.

[4] Lu W, Fadeev AG, Qi BH, Smela E, Mattes BR, Ding J, Spinks GM, Mazurkiewicz J, Zhou DZ, Wallace GG, MacFarlane DR, Forsyth SA, Forsyth M (2002). Use of ionic liquids for pi-conjugated polymer electrochemical devices. Science.

[5] Sun XW, Wang JX (2008). Fast switching electrochromic display using a viologen-modified ZnO nanowire array electrode. Nano Lett.

[6] Zeng Q, McNally A, Keyes TE, Forster RJ (2008). Three colour electrochromic metallopolymer based on a ruthenium phenolate complex bound to poly(4-vinyl)pyridine. Electrochem Commun.

[7] Ling H, Lu J, Phua S, Liu H, Liu L, Huang Y, Mandler D, Lee PS, Lu X (2014). One-pot sequential electrochemical deposition of multilayer poly(3,4-ethylenedioxythiophene): poly (4-styrenesulfonic acid)/tungsten trioxide hybrid films and their enhanced electrochromic properties. J Mater Chem A.

[8] Ziegler JP, Howard BM (1995). Applications of reversible electrodeposition electrochromic devices. Sol Energ Mat Sol C.

[9] Ziegler JP (1999). Status of reversible electrodeposition electrochromic devices. Sol Energ Mat Sol C.

[10] Bechinger C, Ferrer S, Zaban A, Sprague J, Gregg BA (1996). Photoelectrochromic windows and displays. Nature.

[11] Kraft A, Rottmann M (2009). Properties, performance and current status of the laminated electrochromic glass of Gesimat. Sol Energ Mat Sol C.

[12] Bach U, Corr D, Lupo D, Pichot F, Ryan M (2002). Nanomaterials-based electrochromics for paper-quality displays. Adv Mater.

[13] Cho SI, Kwon WJ, Choi SJ, Kim P, Park SA, Kim J, Son SJ, Xiao R, Kim SH, Lee SB (2005). Nanotube-based ultrafast electrochromic display. Adv Mater.

[14] Krebs FC (2008). Electrochromic displays—the new black. Nat Mater.

[15] Monk PMS, Turner C, Akhtar SP (1999). Electrochemical behaviour of methyl viologen in a matrix of paper. Electrochim Acta.

[16] Baloukas B, Lamarre JM, Martinu L (2011). Active metameric security devices using an electrochromic material. Appl Optics.

[17] Cai GF, Tu JP, Zhou D, Li L, Zhang JH, Wang XL, Gu CD (2014). Constructed TiO_2_/NiO core/shell nanorod array for efficient electrochromic application. J Phys Chem C.

[18] Cai GF, Tu JP, Zhou D, Zhang JH, Xiong QQ, Zhao XY, Wang XL, Gu CD (2013). Multicolor electrochromic film based on TiO_2_@Polyaniline core/shell nanorod array. J Phys Chem C.

[19] Cai GF, Zhou D, Xiong QQ, Zhang JH, Wang XL, Tu JP (2013). Efficient electrochromic materials based on TiO_2_@WO_3_ core/shell nanorod arrays. Solar Energy Mater. Solar Cells.

[20] de Mello DAA, Oliveira MRS, de Oliveira LCS, de Oliveira SC (2012). Solid electrolytes for electrochromic devices based on reversible metal electrodeposition. Sol Energ Mat Sol C.

[21] Avellaneda CO, Napolitano MA, Kaibara EK, Bulhoes LOS (2005). Electrodeposition of lead on ITO electrode: influence of copper as an additive. Electrochim Acta.

[22] deTorresi SIC, Carlos IA (1996). Optical characterization of bismuth reversible electrodeposition. J Electroanal Chem.

[23] Imamura A, Kimura M, Kon T, Sunohara S, Kobayashi N (2009). Bi-based electrochromic cell with mediator for white/black imaging. Sol Energ Mat Sol C.

[24] Nakashima M, Ebine T, Shishikura M, Hoshino K, Kawai K, Hatsusaka K (2010). Bismuth electrochromic device with high paper-like quality and high performances. ACS Appl Mater Interfaces.

[25] He Z, Yuan X, Wang Q, Yu L, Zou C, Li C, Zhao Y, He B, Zhang L, Zhang H, Yang H (2016). Multicolored electrochromic device from the reversible aggregation and decentralization of silver nanoparticles. Adv Opt Mater.

[26] Oliveira MRS, Mello DAA, Ponzio EA, de Oliveira SC (2010). KI effects on the reversible electrodeposition of silver on poly(ethylene oxide) for application in electrochromic devices. Electrochim Acta.

[27] Kim TY (2014). Electrochromic device for reversible electrodeposition system. J Inf Display.

[28] Park C, Seo S, Shin H, Sarwade BD, Na J, Kim E (2015). Switchable silver mirrors with long memory effects. Chem Sci.

[29] Krastev I, Valkova T, Zielonka A (2003). Effect of electrolysis conditions on the deposition of silver-bismuth alloys. J Appl Electrochem.

[30] Araki S, Nakamura K, Kobayashi K, Tsuboi A, Kobayashi N (2012). Electrochemical optical-modulation device with reversible transformation between transparent, mirror, and black. Adv Mater.

[31] Tsuboi A, Nakamura K, Kobayashi N (2013). Chromatic characterization of novel multicolor reflective display with electrochemically size-controlled silver nanoparticles. J Soc Inf Display.

[32] Tsuboi A, Nakamura K, Kobayashi N (2013). A localized surface plasmon resonance-based multicolor electrochromic device with electrochemically size-controlled silver nanoparticles. Adv Mater.

[33] Ye T, Xiang Y, Ji H, Hu C, Wu G (2016). Electrodeposition-based electrochromic devices with reversible three-state optical transformation by using titanium dioxide nanoparticle modified FTO electrode. RSC Adv.

[34] Chen HC, Jan DJ, Luo YS, Huang KT (2014). Electrochromic and optical properties of tungsten oxide films deposited with DC sputtering by introducing hydrogen. Appl Optics.

[35] Reichman B, Bard AJ (1979). The electrochromic process at WO_3_ electrodes prepared by vacuum evaporation and anodic oxidation of W. J Electrochem Soc.

[36] Maruyama T, Arai S (1993). The electrochromic properties of nickel-oxide thin-films prepared by chemical vapor deposition. Sol Energ Mat Sol C.

[37] Lu CH, Hon MH, Kuan CY, Leu IC (2014). Preparation of WO_3_ nanorods by a hydrothermal method for electrochromic device. Jpn J Appl Phys.

[38] Park S-I, Quan Y-J, Kim S-H, Kim H, Kim S, Chun D-M, Lee CS, Taya M, Chu W-S, Ahn S-H (2016). A review on fabrication processes for electrochromic devices. Int J Pr Eng Man-G T.

[39] Livage J, Ganguli D (2001). Sol-gel electrochromic coatings and devices: a review. Sol Energ Mat Sol C.

[40] Wang ZC, Hu XF (1999). Fabrication and electrochromic properties of spin-coated TiO_2_ thin films from peroxo-polytitanic acid. Thin Solid Films.

[41] Deepa M, Singh P, Sharma SN, Agnihotry SA (2006). Effect of humidity on structure and electrochromic properties of sol-gel-derived tungsten oxide films. Sol Energ Mat Sol C.

[42] Sun X, Cao H, Liu Z, Li J (2009). Influence of annealing temperature on microstructure and optical properties of sol-gel derived tungsten oxide films. Appl Surf Sci.

[43] Deepa M, Saxena TK, Singh DP, Sood KN, Agnihotry SA (2006). Spin coated versus dip coated electrochromic tungsten oxide films: structure, morphology, optical and electrochemical properties. Electrochim Acta.

[44] Deepa M, Sharma R, Basu A, Agnihotry SA (2005). Effect of oxalic acid dihydrate on optical and electrochemical properties of sol-gel derived amorphous electrochromic WO_3_ films. Electrochim Acta.

[45] Kim SK, Cho CH, Kim BH, Choi YS, Park SJ, Lee K, Im S (2009). The effect of localized surface plasmon on the photocurrent of silicon nanocrystal photodetectors. Appl Phys Lett.

[46] Rao SG, Gondal MA, Dastageer MA (2013). Thickness dependent morphology of Au and TiO_2_ and optical study of TiO_2_ thin films on patterns of self-assembled monolayers. Surf Coat Technol.

[47] van Ginneken B, Stavridi M, Koenderink JJ (1998). Diffuse and specular reflectance from rough surfaces. Appl Opt.

[48] Niu W, Wang G, Liu XD, Tang J, Bi XG (2015). Preparation of WO_3_-TiO_2_ photo-anode and its performance of photocatalytic hydrogen production by water splitting. Int J Electrochem Sc.

[49] Yao DD, Rani RA, O’Mullane AP, Zadeh KK, Ou JZ (2014). Enhanced coloration efficiency for electrochromic devices based on anodized Nb_2_O_5_/electrodeposited MoO_3_ binary systems. J Phys Chem C.

[50] Zhang J, Tu JP, Cai GF, Du GH, Wang XL, Liu PC (2013). Enhanced electrochromic performance of highly ordered, macroporous WO_3_ arrays electrodeposited using polystyrene colloidal crystals as template. Electrochim Acta.

[51] Yang LL, Ge DT, Zhao JP, Ding YB, Kong XP, Li Y (2012). Improved electrochromic performance of ordered macroporous tungsten oxide films for ir electrochromic device. Sol Energ Mat Sol C.

[52] Kim YJ, Jeong HK, Seo JK, Chai SY, Kim YS, Lim GI, Cho MH, Lee IM, Choi YS, Lee WI (2007). Effect of TiO_2_ particle size on the performance of viologen-anchored TiO_2_ electrochromic device. J Nanosci Nanotechnol.

[53] Esmail A, Hashem H, Soltan S, Hammam M, Ramadan A (2017). Thickness dependence of electro-optical properties of WO_3_ films as an electrochromic functional material for energy-efficient applications. Phys Status Solidi A.

[54] Sun X, Liu Z, Cao H (2010). Effects of film density on electrochromic tungsten oxide thin films deposited by reactive dc-pulsed magnetron sputtering. J Alloys Compd.

[55] Zhou JL, Luo G, Wei YX, Zheng JM, Xu CY (2015). Enhanced electrochromic performances and cycle stability of NiO-based thin films via Li-Ti co-doping prepared by sol-gel method. Electrochim Acta.

[56] Ren Y, Chim WK, Guo L, Tanoto H, Pan JS, Chiam SY (2013). The coloration and degradation mechanisms of electrochromic nickel oxide. Sol Energ Mat Sol C.

