# Supplementary material for: Dip-Coating Process Engineering and Performance Optimization for Three-State Electrochromic Devices
Source: Nanoscale Res Lett. 2017 Jun 6;12:390. doi: 10.1186/s11671-017-2163-0 (PMC5461225; doi:10.1186/s11671-017-2163-0)
Supplement: Additional file 1:Figure S1. — (Color online) XRD patterns of dip-coated TiO2 thin film (dip-coated on the FTO electrode and sintered at 500 °C for 30 min), spin-coated TiO2 thin film (spin-coated on the FTO electrode and sintered at 500 °C for 30 min), fresh TiO2 nanoparticles (purchased and untreated), flat FTO electrode (cleaned and dried). (a) 5~10, (b) 40, and (c) 100 nm, respectively. Figure S2. (Color online) Photographs of TiO2 thin films after Ag deposition with nanoparticle sizes of (a) 5~10, (b) 40, and (c) 100 nm, respectively. In-plane SEM images of TiO2 thin films after Ag deposition with nanoparticle sizes of (d) 5~10, (e) 40, and (f) 100 nm, respectively. Cross-sectional SEM images of TiO2 thin films after Ag deposition with nanoparticle sizes of (g) 5~10, (h) 40, and (i) 100 nm, respectively. Figure S3. (Color online) SEM images of dip-coated TiO2 thin film after switching between its coloration and bleached states for 1500 cycles. Figure S4. (Color online) Optical properties of the electrodeposition-based electrochromic device in transparent (red), black (blue), and mirror states (green). Reflectance spectra of modified devices prepared with different fabrication conditions, including (a) lifting speed of 2000 μm/s, (b) lifting speed of 1000 μm/s, (c) precursor concentration of 1:3, (d) precursor concentration of 1:4, (e) dipping number of 3, and (f) dipping number of 5. Table S1. Coloration efficiency (CE) of the electrochromic devices modified with different modification parameters. Table S2. Element ratios of TiO2 thin film prepared with 100 nm TiO2 nanoparticle after 1500 cycles. (DOCX 7367 kb) [file 11671_2017_2163_MOESM1_ESM.docx]

Additional file (ESI) for Nanoscale Research Letters

Dip-coating Process Engineering and Performance Optimization for Three-state Electrochromic Devices

**Additional file**

L. Wu,^a^ D.J. Yang,^a^ L.X. Fei,^a^ Y. Huang,^a^ F. Wu,^a^ Y.L. Sun,^a^ J.Y. Shi,^a^ and Y. Xiang*^a^

*^a^ School of Energy Science and Engineering, University of Electronic Science and Technology of China, Chengdu, Sichuan, P. R. China.*

**E-mail: xyg@uestc.edu.cn (Y. Xiang); Tel & Fax: +86 28 6183 1556*

**Figure S1**^†^


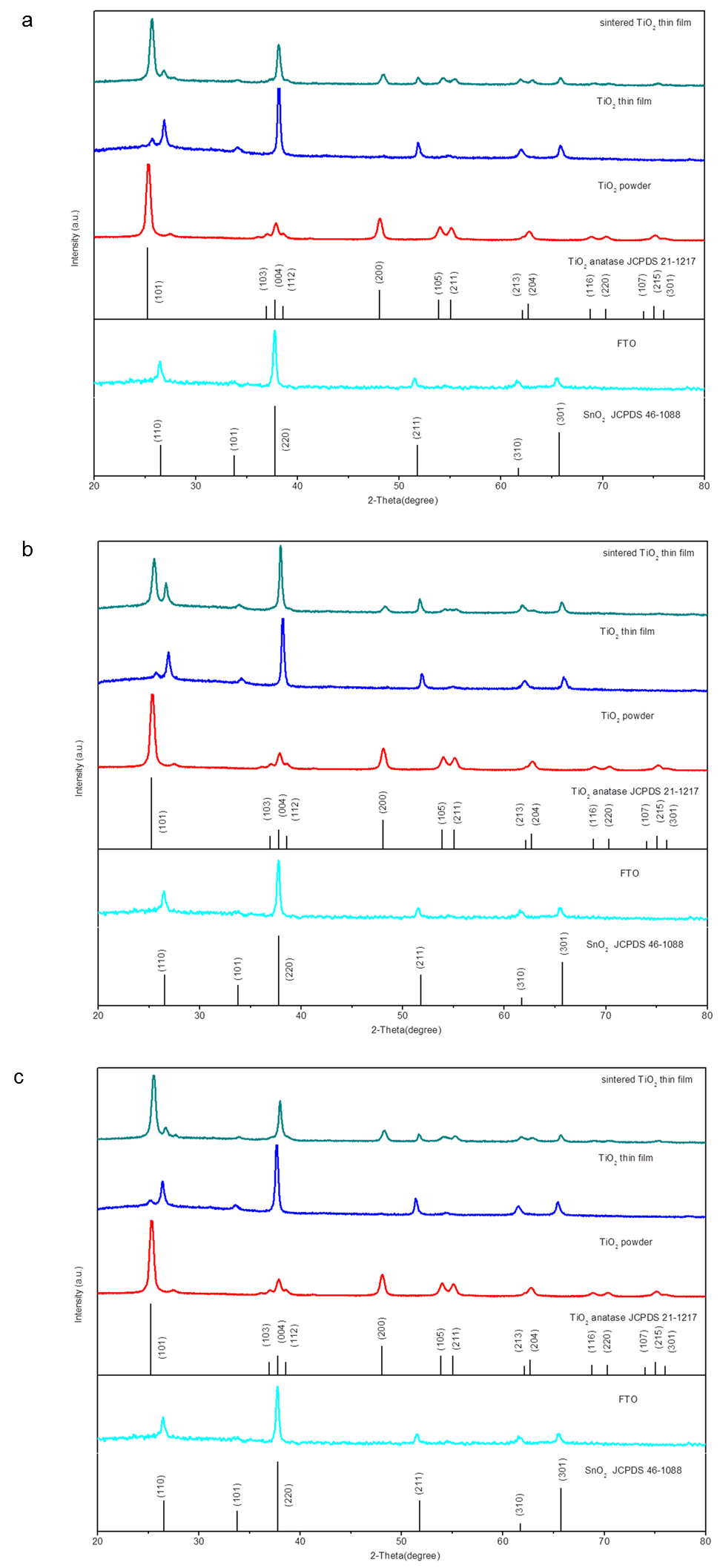


Figure S1^†^

(Lu Wu et al.)

**Figure S1†**.(Color online) XRD patterns of dip-coated TiO_2_ thin film (dip-coated on the FTO electrode and sintered at 500 °C for 30 min), spin-coated TiO_2_ thin film (spin-coated on the FTO electrode and sintered at 500 °C for 30 min), fresh TiO_2_ nanoparticles (purchased and untreated), flat FTO electrode (cleaned and dried). (a) 5~10 nm, (b) 40 nm, and (c) 100 nm, respectively.

**Figure S2**^†^


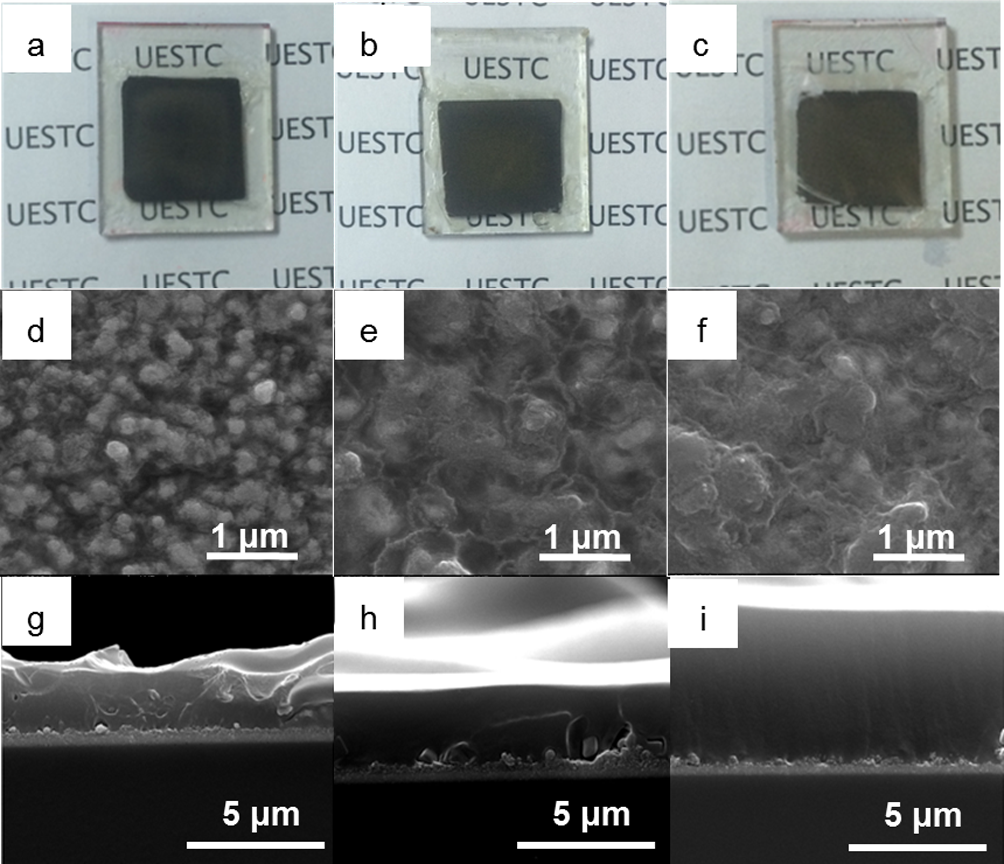


Figure S2^†^

(Lu Wu et al.)

**Figure S2†**.(Color online) Photographs of TiO_2_ thin films after Ag deposition with nanoparticle size of (a) 5~10 nm, (b) 40 nm, and (c) 100 nm respectively. In-plane SEM images of TiO_2_ thin films after Ag deposition with nanoparticle size of (d) 5~10 nm, (e) 40 nm, and (f) 100 nm respectively. Cross-sectional SEM images of TiO_2_ thin films after Ag deposition with nanoparticle size of (g) 5~10 nm, (h) 40 nm, and (i) 100 nm respectively.

**Figure S3**^†^


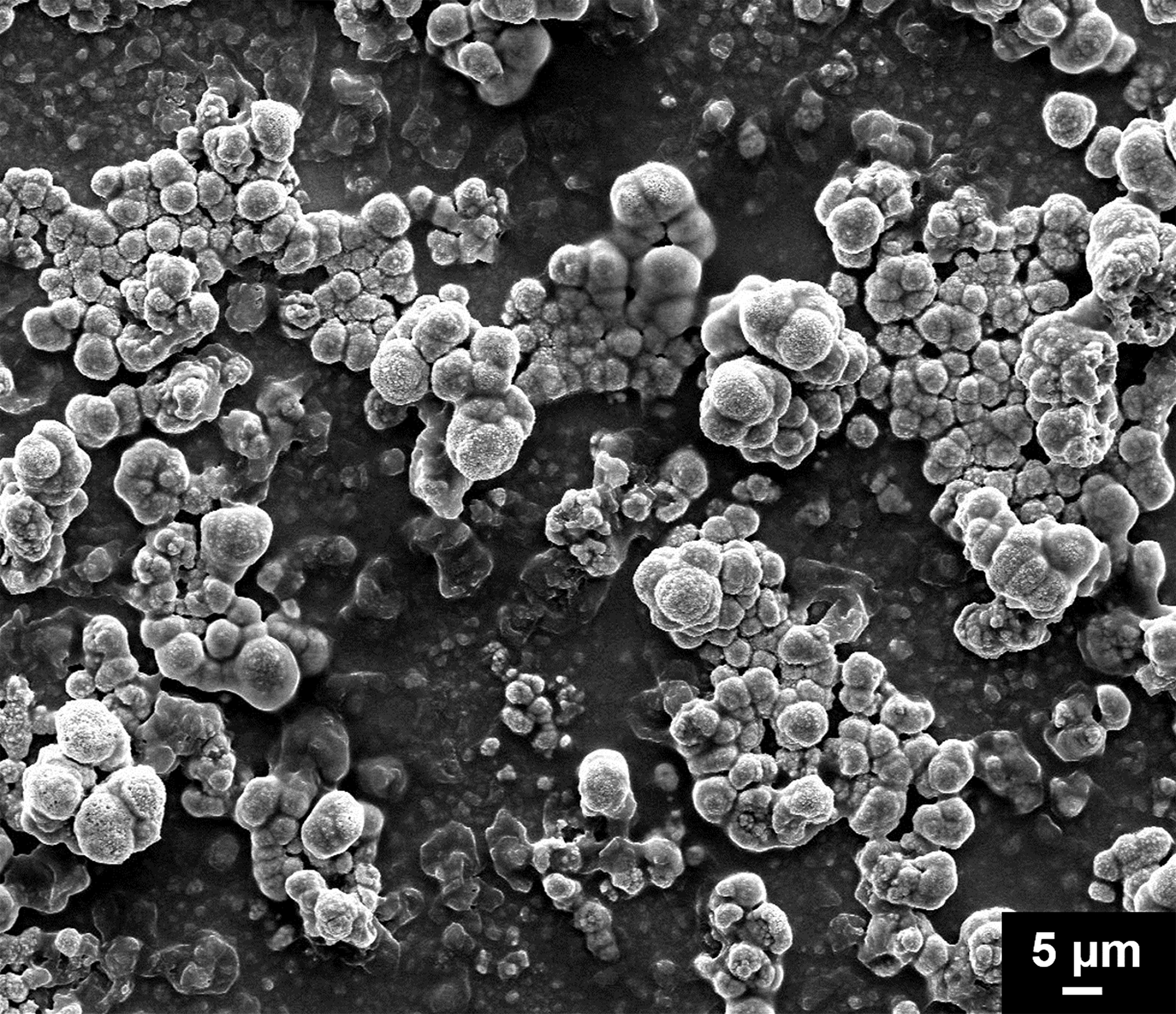


Figure S3^†^

(Lu Wu et al.)

**Figure S3†**.(Color online) SEM images of dip-coated TiO_2_ thin film after switching between its coloration and bleached states for 1500 cycles

**Figure S4**^†^

**
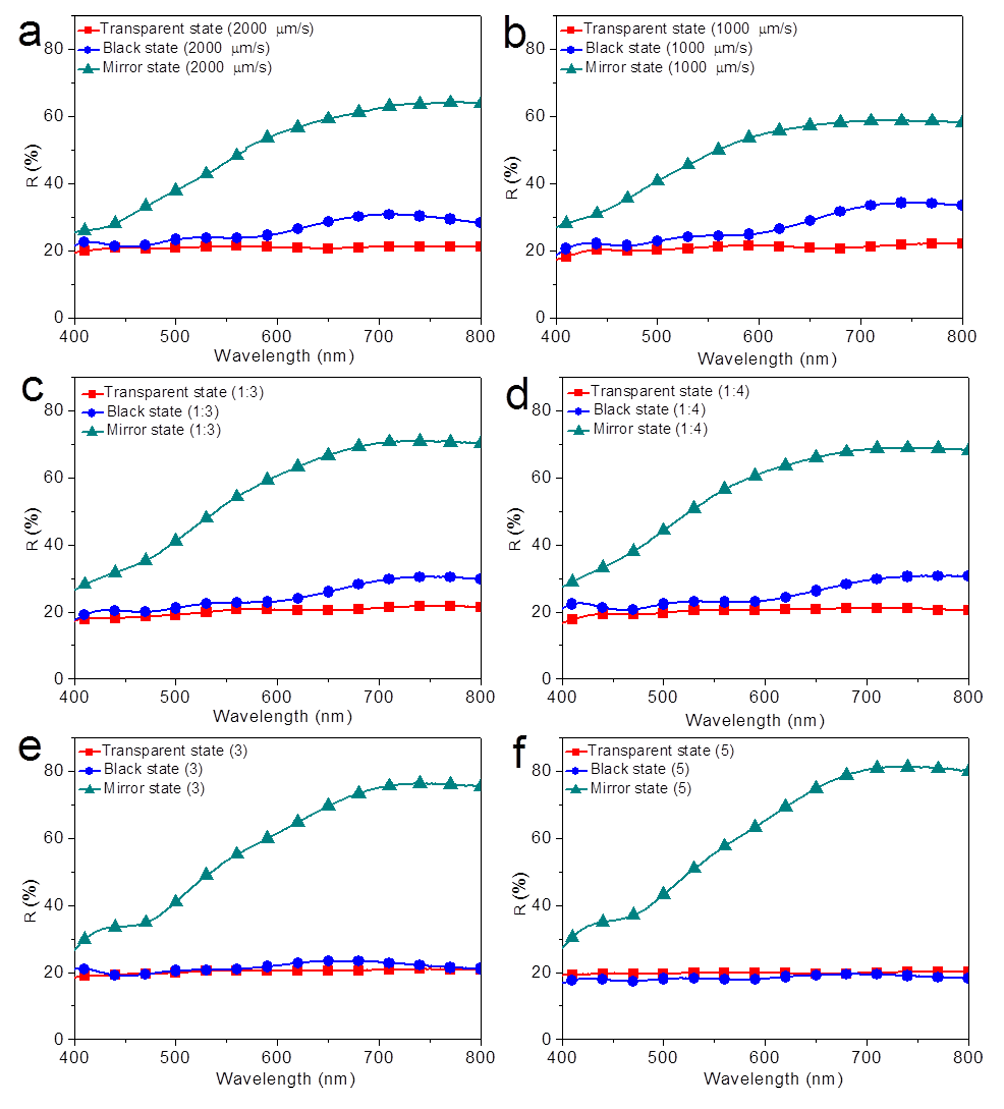
**

Figure S4^†^

(Lu Wu et al.)

**Figure S4**^†^**.** (Color online) Optical properties of the electrodeposition-based electrochromic device in transparent (red), black (blue), and mirror states (green). Reflectance spectra of modified devices prepared with different fabrication conditions, including (a) lifting speed of 2000 μm/s, (b) lifting speed of 1000 μm/s, (c) precursor concentration of 1:3, (d) precursor concentration of 1:4, (e) dipping number of 3, (f) dipping number of 5.

**Table S1**^†^

| Table S1† Coloration efficiency (CE) of the electrochromic devices modified with different modification parameters | | |
| --- | --- | --- |
| 5~10 nm | 27.0 cm^2^/C |  |
| 40 nm | 20.7 cm^2^/C |  |
| 100 nm | 16.9 cm^2^/C |  |
| 2000 μm/s | 30.0 cm^2^/C |  |
| 1000 μm/s | 32.6 cm^2^/C |  |
| 1:3 | 31.7 cm^2^/C |  |
| 1:4 | 34.0 cm^2^/C |  |
| N=3 | 20.2 cm^2^/C |  |
| N=5 | 11.2 cm^2^/C |  |

(Lu Wu et al.)

**Table S2**^†^

| Table S2† Elements ratios of TiO_2_ thin film prepared with 100 nm TiO_2_ nanoparticle after 1500 cycles | | |
| --- | --- | --- |
| O | 18.90% |  |
| Ti | 4.67% |  |
| Ag | 32.06% |  |
| Sn | 44.37% |  |

(Lu Wu et al.)
